# THE ECONOMIC CONSEQUENCES OF GRAY DIVORCE FOR WOMEN AND MEN

**DOI:** 10.1093/geroni/igac059.1171

**Published:** 2022-12-20

**Authors:** I-Fen Lin, Susan Brown

**Affiliations:** Bowling Green State University, Bowling Green, Ohio, United States; Bowling Green State University, Bowling Green, Ohio, United States

## Abstract

Surprisingly little is known about the consequences of gray divorce (after age 50) and how women and men fare economically during the aftermath. Using longitudinal data from the 2004-2014 Health and Retirement Study, we estimated hybrid fixed/random effects models comparing women’s and men’s economic well-being prior to, during, and following gray divorce and subsequent repartnering. Women experienced a 45% decline in their standard of living, whereas men’s dropped by just 21%. These declines persisted over time for men, and only reversed for women following repartnering, which essentially offset women’s losses associated with gray divorce. Both men and women experienced roughly a 50% drop in wealth. Although repartnering seems to reverse most of the economic costs of gray divorce for women, few form new co-residential unions after divorce. This study offers insight about the financial aftermath of gray divorce, which is likely to contribute to growing economic disadvantage among older adults.

